# Genomic Characterization of the *Taylorella* Genus

**DOI:** 10.1371/journal.pone.0029953

**Published:** 2012-01-03

**Authors:** Laurent Hébert, Bouziane Moumen, Nicolas Pons, Fabien Duquesne, Marie-France Breuil, Didier Goux, Jean-Michel Batto, Claire Laugier, Pierre Renault, Sandrine Petry

**Affiliations:** 1 ANSES, Dozulé Laboratory for Equine Diseases, Dozulé, France; 2 Institut National de la Recherche Agronomique, UMR1319 Micalis, Domaine de Vilvert, Jouy-en-Josas, France; 3 Centre de Microscopie Appliquée à la Biologie, Université de Caen Basse-Normandie et IFR146 ICORE, Caen, France; Emory University, United States of America

## Abstract

The *Taylorella* genus comprises two species: *Taylorella equigenitalis*, which causes contagious equine metritis, and *Taylorella asinigenitalis,* a closely-related species mainly found in donkeys. We herein report on the first genome sequence of *T. asinigenitalis*, analyzing and comparing it with the recently-sequenced *T. equigenitalis* genome. The *T. asinigenitalis* genome contains a single circular chromosome of 1,638,559 bp with a 38.3% GC content and 1,534 coding sequences (CDS). While 212 CDSs were *T. asinigenitalis*-specific, 1,322 had orthologs in *T. equigenitalis.* Two hundred and thirty-four *T. equigenitalis* CDSs had no orthologs in *T. asinigenitalis*. Analysis of the basic nutrition metabolism of both *Taylorella* species showed that malate, glutamate and alpha-ketoglutarate may be their main carbon and energy sources. For both species, we identified four different secretion systems and several proteins potentially involved in binding and colonization of host cells, suggesting a strong potential for interaction with their host. *T. equigenitalis* seems better-equipped than *T. asinigenitalis* in terms of virulence since we identified numerous proteins potentially involved in pathogenicity, including hemagluttinin-related proteins, a type IV secretion system, TonB-dependent lactoferrin and transferrin receptors, and YadA and Hep_Hag domains containing proteins. This is the first molecular characterization of *Taylorella* genus members, and the first molecular identification of factors potentially involved in *T. asinigenitalis* and *T. equigenitalis* pathogenicity and host colonization. This study facilitates a genetic understanding of growth phenotypes, animal host preference and pathogenic capacity, paving the way for future functional investigations into this largely unknown genus.

## Introduction

*Taylorella equigenitalis* is a Gram-negative coccobacillus, classified in the *Alcaligenaceae* family [1]. It is the causative agent of contagious equine metritis (CEM), a sexually-transmitted infection of horses first reported in 1977 [2], [3], and currently detected in many countries and various horse breeds. Notified to the OIE (World Organisation for Animal Health), CEM is characterized in infected mares by abundant mucopurulent vaginal discharge and a variable degree of vaginitis, endometritis and cervicitis, usually resulting in temporary infertility [4]. In stallions, no clinical signs are observed, and asymptomatic carrier mares have also been reported [5]. CEM is usually transmitted by sexual contact with asymptomatic carrier stallions. Indirect genital contact between an infected mare and a stallion (or vice versa) is also an important factor in the spread of CEM, since infective semen and indirect venereal contact through the use of contaminated fomites such as vaginal specula, artificial vaginas, wash buckets or tail bandages can disseminate the infection [4].

In terms of biochemical properties, the *T. equigenitalis* genus has fastidious growth requirements and is dependent on enriched bacteriologic media and microaerophilic incubation conditions to grow. This bacterium has been reported to be independent of glycolysis and hexose monophosphate pathways and dependent on tricarboxylic acid (TCA) cycle and oxidative phosphorylation for cell energy [6]. *In vitro* and *in vivo* morphological studies have shown that *T. equigenitalis* has a capsule [7] and expresses pili *in vivo* [8]. *T. equigenitalis* remains able to replicate in equine neutrophils [9] and has been described as having invasive and replicative abilities through an equine derm cell invasion assay [10]. To date, no precise virulence factor has been reported for *T. equigenitalis*.

Until the discovery of new bacterial isolates from two jacks and a mare with no clinical signs in 1997–1998 [11], the *Taylorella* genus consisted of only one species. This newly-identified bacterium, characterized by a slight difference in colony morphology, a notably slower growth rate and divergent immunofluorescence characteristics compared to T. *equigenitalis*, has been classified following taxonomic studies as a new species named *Taylorella asinigenitalis* [12]. Due to their high degree of relatedness, it remains difficult to differentiate the two *Taylorella* species using classical identification techniques. There have already been reports of *T. asinigenitalis* being incorrectly identified as *T. equigenitalis* [13]. To date, only the detection of *T. equigenitalis* in a horse leads to the declaration of CEM. However, the question of whether to declare a case of CEM following infection by *T. asinigenitalis* remains relevant since it has been reported that mares experimentally infected with *T. asinigenitalis* could develop clinical signs of metritis and cervicitis [11].

In order to understand what differentiates the two closely-related *Taylorella* species, particularly in terms of metabolism and virulence capacity, we herein report the first genome sequence of *T. asinigenitalis* and carry out a comparative genomic analysis between this sequence and the recently-described genome sequence of *T. equigenitalis* [14].

## Results

### *T. asinigenitalis* and *T. equigenitalis* genome properties and general features

**Figure 1:**
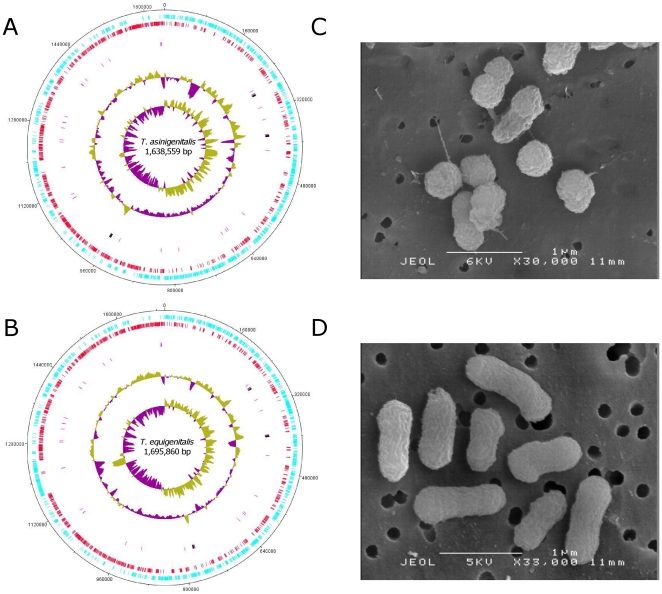
Circular representation of the *T. asinigenitalis* MCE3 and *T. equigenitalis* MCE9 genomes. (A and B) Circular representation of *T. asinigenitalis* MCE3 chromosome and *T. equigenitalis* MCE9 chromosome, respectively. The outer circle shows position in bp. The second (blue) and third circles (red) show forward CDSs and reverse CDSs, respectively. The fourth circle shows rRNA (black) and tRNA (pink). The fifth circle shows the G+C% content plot. The innermost circle shows GC skew, purple indicating negative values and olive, positive values (the replication origins are clearly detectable). (C and D) Scanning electron micrographs of *T. asinigenitalis* MCE3 observed at x30,000 and of *T. equigenitalis* MCE9 observed at x33,000, respectively. Cells were observed in a stationary growth phase. The major divergence observed by transmission electron microscopy analysis of *T. asinigenitalis* and *T. equigenitalis* is a coccoid shape for *T. asinigenitalis* and a rode shape for *T. equigenitalis*. However, more studies are necessary to confirm this observation since it has been shown that *T. equigenitalis* could harbor bacillary or coccoid forms within a single colony [7].

**Figure 2:**
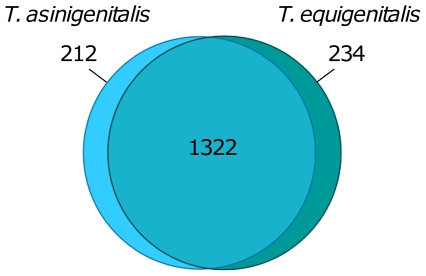
Venn diagram illustrating the number of putative proteins associated with *T. asinigenitalis* and *T. asinigenitalis*. The diagram was constructed by reciprocal FASTA using a minimum cutoff of 50% amino acid similarity over 80% or more of the sequence and the surfaces are proportional to the number of genes.

*T. asinigenitalis* (Figure 1A and 1C) has a single 1,638,559 bp circular chromosome with an overall G+C content of 38.3%, containing 1,534 coding sequences (CDSs), 9 rRNA genes, 38 tRNA genes (Table 1 and Figure 1A). No plasmid was found. We identified 1,534 protein-coding genes with an average length of 987 bp corresponding to a protein-coding content of 92.4%. Of these, 1,231 (≈79%) genes were assigned a predicted function. Table 1 presents both *T. asinigenitalis* and the previously-described *T. equigenitalis* genome characteristics (Figure 1B and 1D) [14]. According to GC skew analysis [(G−C)/(G+C)], the likely origin of replication of the *T. asinigenitalis* and *T. equigenitalis* chromosome and the replication termination site of the chromosome which appears diametrically opposed to the origin can be consistently proposed (Figure 1A and 1B). Direct comparisons between the predicted CDSs of *T. asinigenitalis* and *T. equigenitalis* were performed by reciprocal FASTA using a minimum cutoff of 50% amino acid similarity over 80% of their length or more. The results revealed that about 1,322 CDSs (86.18% and 84.96% of the total genes predicted in *T. asinigenitalis* and *T. equigenitalis* respectively) are common to both *Taylorella* species (Figure 2). The average nucleotide identity of the genes common to both strains is 79.1%, and the average amino acid identity 73.7%. Moreover, we identified 212 *T. asinigenitalis* sequences that gave no hits or non-significant hits in *T. equigenitalis* (Table S1), and reciprocally, 234 of *T. equigenitalis* absent in *T. asinigenitalis* (Table S2).

**Table 1:** General features of the genomes of *T. asinigenitalis* MCE3 and *T. equigenitalis* MCE9.

	Size (bp)	GC%	No. of CDSs	coding (%)	Coding density (average CDS length in bp)	rRNA clusters	tRNAs	Insertion elements^a^	Plasmid
*T. asinigenitalis* MCE3	1,638,559	38.3	1,534	92.4	0.936 (987)	3	38	0	0
*T. equigenitalis* MCE9	1,695,860	37.4	1,556	92.5	0.918 (1090)	3	38	0	0

a According to IS Finder (http://www-is.biotoul.fr/is.html), E value>0.053 and>0.11 for *T. asinigenitalis* MCE3 and *T. equigenitalis* MCE9, respectively.

### Burkholderiales phylogeny

**Figure 3:**
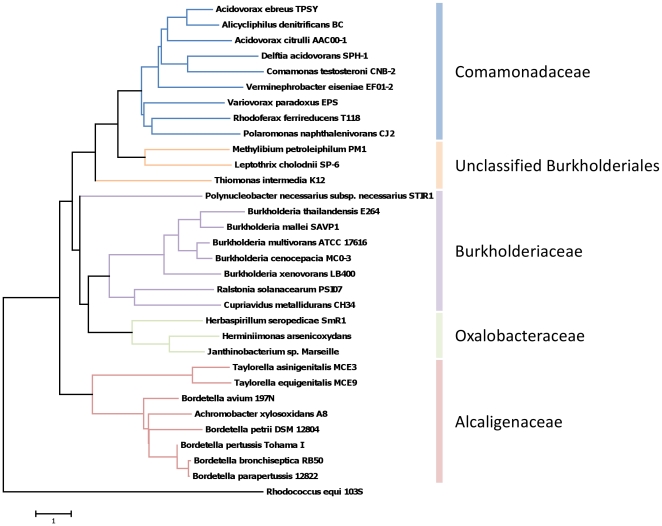
Phylogenomic analysis of 31 representative Burkholderiales species. We selected a set of 31 genomes belonging to the Burkholderiales order and one outgroup genome (*Rhodococcus equi* 103S) (Table S3). Putative core ortholog genes were identified by using Scissors implemented in the iMOMi framework [15]. A similarity distance matrix was built with the average percentage of amino acid sequence similarity provided by CLUSTALW [80] comparisons (distance = 100 - average percent similarity) and used to infer a neighbor-joining tree with MEGA 5.05 software [81].

Burkholderiales phylogeny was constructed based on putative core ortholog genes of 31 bacterial genome sequences of the Burkholderiales order identified by using Scissors implemented in the iMOMi framework [15]. The results of our phylogenomic analyses (Figure 3) are consistent with previously described 16S rRNA-derived β-proteobacteria phylogenies [16] and shows that the two *Taylorella* species are markedly distant from the other members of Alcaligenaceae.

### Genome alignment

**Figure 4:**
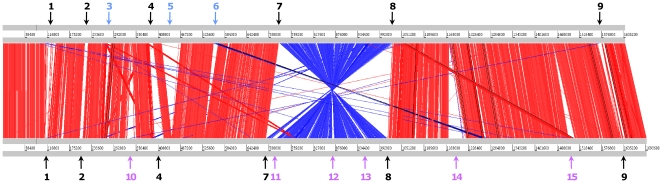
Sequence alignment of *T. asinigenitalis* MCE3 and *T. equigenitalis* MCE9 genomes. The genomes of *T. asinigenitalis* MCE3 and *T. equigenitalis* MCE9 were compared using BLASTN [82] and these comparisons visualized with the Artemis comparison tool (ACT) [83]. Red and blue lines connect homologous regions in direct and reverse orientation, respectively. Gaps indicate unique DNA. The gray bars represent forward and reverse strands. Blue region numbers and arrows indicate *T. asinigenitalis* MCE3-specific regions, pink region numbers and arrows indicate *T. equigenitalis* MCE9-specific regions, and black region numbers and arrows indicate variable regions. Rearrangement regions and strain-specific regions are briefly described in table 2 and analyzed in detail in Additional files 1 and 2.

The Artemis comparison tool (ACT) was used to examine the conservation of gene order (synteny) between *T. asinigenitalis* and *T. equigenitalis* genomes (Figure 4). The alignment shows that the vast majority of common genes are in the same order. The average identity of the aligned portion of the genomes (>100 bp) is 83.3%. Despite the globally conserved synteny, genome alignment revealed a large chromosomal inversion symmetrical across the replication axis and 21 major divergent genomic regions, described in Table 2 and below. To determine if this inversion is conserved in *T. asinigenitalis* and *T. equigenitalis* strains, we constructed PCR primer sets based on the genome sequences of *T. asinigenitalis* and *T. equigenitalis* (Table S3) to determine by long PCR analysis the orientation of the large inverted genomic regions for 30 *T. asinigenitalis* and 30 *T. equigenitalis* strains (Table S4). The results of these experiments revealed that the orientation of this large genomic fragment is specifically conserved in each species. Given that the chromosomal inversion in *T. asinigenitalis* compared to *T. equigenitalis* is symmetrical across the replication axis, this event did not change gene orientation with respect to the replication axis and thus did not lead to any GC skew discontinuity.

**Table 2:** Main characteristics of divergent regions between *T. asinigenitalis* MCE3 and *T. equigenitalis* MCE9 genomes.

	*T. asinigenitalis* MCE3			*T. equigenitalis* MCE9		
Region No^a^	Hypothetical function^b^	Length (kbp)	No of specific gene	Hypothetical function	Length (kbp)	No of specific gene
1	Region of unknown function	33.1	30	Region of unknown function	5.5	5
2	CRISPR-associated region	11.4	5	Restriction modification	10.6	9
4	Antigen O region	9.0	5	Antigen O region	20.5	12
7	Restriction Modification	3.0	2	Inversion/rearrangement region	1.4	3
8	Inversion/rearrangement region	7.9	8	Inversion/rearrangement region	9.0	5
9	Region of unknown function	10.0	4	YadA and Hep_Hag related region	3.1	3
3	Region of unknown function	9.2	3			
5	Prophage region	50.9	64			
6	Region of unknown function	2.4	5			
10				Hemagluttinin related region	25.4	5
11				ABC transporter related region	14.0	11
12				Efflux system	5.8	3
13				Region of unknown function	4.6	5
14				Type IV secretion system	45.6	57
15				Rhs related region	15.2	10

a Numbered regions correspond to the regions shown in Figure 4.

b Determined on the basis of sequence homology.

### Strain-specific genes and regions in *T. asinigenitalis* and *T. equigenitalis*

Strain-specific regions in *T. asinigenitalis* and *T. equigenitalis* were determined using ACT software and then specific regions in each genome were manually curated. These strain-specific regions were numbered and their position shown in Figure 4, summarized in Table 2 and detailed in Additional files 1 and 2.

The *T. asinigenitalis* genome contains three specific regions (Regions 3, 5 and 6; Figure 4 and Table S2) composed of 72 CDSs. Furthermore, 86 *T. asinigenitalis*-specific CDSs mainly composed of hypothetical proteins (Table S2) are randomly inserted into the genome. Regions 3 and 6 are composed of (i) three hypothetical proteins, and (ii) four hypothetical proteins and DnaJ, a hypothetical chaperone protein, respectively (Table S2). Region 5 is the largest *T. asinigenitalis*-specific region, with 64 specific CDSs (TASI_0412 – TASI_0476) distributed over a genomic region of 50.9 kb (Figure 4 and Table S2). This region was assigned as a putative prophage, while no prophage element could be identified in the *T. equigenitalis* genome.

The *T. equigenitalis* genome contains six strain-specific regions (Regions 10–15; Figure 4 and Table S1) containing 91 unique CDSs; another 106 *T. equigenitalis*-specific CDSs are distributed over the *T. equigenitalis* genome (Table S1). Region 10 encodes five proteins, two of which are annotated as hemagglutinin, able to induce the agglutination of erythrocytes and thus to be involved in virulence in the phylogenetically related *Bordetella* genus [17]. This region has therefore been classified as a hemagglutinin-related region (Table 2). Region 11 encodes six hypothetical proteins and five proteins potentially involved in transmembrane transport, including three ABC transporter-related proteins. It was thus classified as an ABC transporter-related region. Region 12 contains three putative efflux system transmembrane RND (Resistance-Nodulation-cell Division) proteins, previously determined as being involved in virulence and resistance to antimicrobial compounds [18]. Region 13, composed of four hypothetical proteins and a protein containing a relaxase domain (pfam03432) potentially involved in the horizontal transfer of genetic information, has been classified as a region of unknown function. Region 14 is the longest *T. equigenitalis*-specific region, with 57 specific CDSs. It encodes type IV secretion system (T4SS). These systems are membrane-associated transporter complexes used by various bacteria to deliver substrate molecules to a wide range of target cells and are in particular involved in toxin secretion and the injection of virulence factors into eukaryotic host target cells by several mammalian pathogens as described below [19]. Region 15 was classified as a rearrangement hot-spot (Rhs)-related region. It is composed of 10 CDSs including three Rhs-family proteins which contain a repeated motif and are potentially located on the cell surface. Rhs-like elements have been determined as being involved in bacteriocin production in *Pseudomonas savastanoi* pv. *Savastanoi* [20].

Of the 15 regions specific to the *T. asinigenitalis* and *T. equigenitalis* genomes, six (Regions 1, 2, 4, 7, 8 and 9) exist in both strains but have different gene contents and are designated as variable regions.

For both species, region 1 is composed of hypothetical proteins only, and must therefore be classified as a region of unknown function.

**Figure 5:**
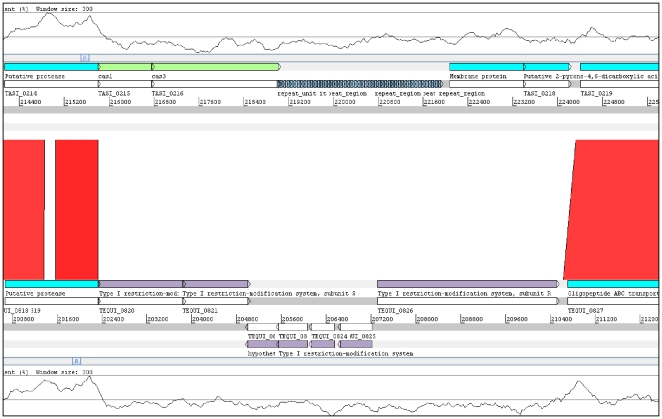
Comparative analysis of *T. asinigenitalis* MCE3 CRISPR and *T. equigenitalis* MCE9 R/M system associated loci. The *T. asinigenitalis* MCE3 CRISPR-associated locus and the *T. equigenitalis* MCE9 R/M system locus were compared using BLASTN [82] and comparisons were visualized with ACT [82]. Red lines connect homologous regions in direct orientation. Gaps indicate unique DNA. The gray bars represent forward and reverse strands. Repeats are shown in dark blue, *cas* genes appear in green, and neighboring open reading frames are light blue. The GC content plot (300 bp window) is shown at the top and bottom of the Figure. This region corresponds to a detailed view of region 2 in Figure 4.

Region 2, detailed in Figure 5, is composed of clustered regularly interspaced short palindromic repeats (CRISPRs) and restriction/modification (R/M) systems in *T. asinigenitalis* and *T. equigenitalis* respectively. CRISPR and R/M systems mediate resistance to infection by foreign DNA genetic material (e.g., bacteriophages, conjugative plasmids and transposable elements) and thus inhibit horizontal gene transfer [21]. The *T. asinigenitalis* CRISPR system contains two genes very similar to the cas1 and cas3 genes: TASI_0215 and TASI_0216 (BLAST E value<E-100) (Figure 5) and has 49 direct repeats of 28 bp each with 48 spacer sequences 33 bp long. By BLAST analysis, one of these spacer sequences appeared to be homologous to a prophage sequence and one to a genome part of the intracellular pathogen *Rickettsia bellii* (Table S5). No sequence homology was found for any other spacer sequences. As already described for E. coli and *Sulfolobus sulfataricus* [22], no leader sequences were found in *T. asinigenitalis* CRISPR loci and no mobile element was found near the CRISPR locus. In the corresponding *T. asinigenitalis* CRISPR cluster locus, *T. equigenitalis* genome displays six genes related to type I restriction-modification systems (Figure 5 and Table S1). R/M systems are composed of genes encoding a restriction enzyme and a modification methylase. They act as a defense mechanism protecting the host bacterium from invaders by attacking non-self DNA [23]. Interestingly, although the content of this region diverges in each *Taylorella* species, each locus nonetheless contains a mechanism for defense against invaders by attacking non-self DNA.

Region 4 in both *T. asinigenitalis* and *T. equigenitalis* is composed of genes involved in lipopolysaccharide (LPS) O-antigen biosynthesis. By their capacity to induce an intense host immune response, LPS O-antigens could be considered potential virulence-related factors [24]. The structural analysis of the LPS produced by *T. asinigenitalis* ATCC 700933 and *T. equigenitalis* ATCC 35865 shows that the O-antigen structure of each species differs by its chemical structure and serological characteristics [24], [25]. This observation is consistent with the fact that the LPS O-antigen has been used as a specific marker for identifying and differentiating *T. equigenitalis* and *T. asinigenitalis* [26]. Characterizing the chromosomal region encoding the LPS O-antigen for *T. asinigenitalis* and *T. equigenitalis* may allow the finding of still unknown O-antigen variants and foster the development of specific detection assays to distinguish the two species.

Region 7, located at the edge of the large inverted regions of the genome, contains one putative restriction/modification (R/M) system composed of a restriction enzyme and a DNA-cytosine methyltransferase in *T. asinigenitalis* and three hypothetical proteins in *T. equigenitalis*. The presence of a R/M locus at the edge of these large inverted regions, already described for different genomes [27], [28], and were proposed to be involved in genomic inversion [23].

Region 8 in *T. asinigenitalis* includes seven hypothetical proteins and a tripartite ATP-independent periplasmic transporter (TRAP); the content of this region in *T. equigenitalis* is quite different and includes four hypothetical proteins and an outer membrane receptor protein.

Region 9 in *T. asinigenitalis* contains three hypothetical proteins encoding genes and one potential ATP-dependent DNA helicase RecG, which plays a critical role in recombination and DNA repair [29]. Region 9 in *T. equigenitalis* includes one YadA-like-protein, one Hep_Hag family protein and one hypothetical protein. Proteins containing YadA and Hep_Hag domains have been associated with autotransporters and share protein domain architectures with hemagglutinins and invasions. They have been shown to have an important virulence-related role by acting as multimeric surface proteins that modulate cell interactions with the host and environment [30]. As an example, *Burkholderia mallei* proteins containing YadA and Hep_Hag domains have been shown to generate a strong antibody response in the experimental equine model of glanders [30].

Generally speaking, the presence of only a few locus rearrangements between the two genomes suggests a recent speciation which is consistent with the close phylogenomic relationship of these two species as shown in the Burkholderiales phylogenomic tree presented in Figure 3.

### Basic nutrition metabolism

**Figure 6:**
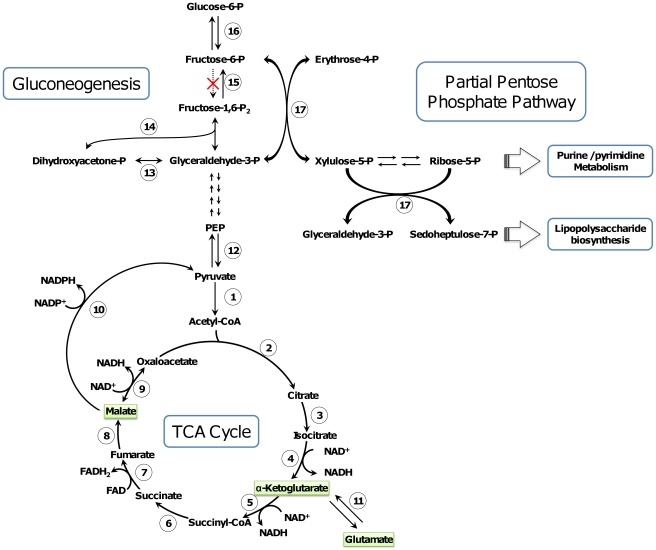
Proposed carbon flux in the *Taylorella* genus. Enzymes participating in major reactions are numbered thus: 1. pyruvate dehydrogenase (TASI_0903, TEQUI_1387); 2. Citrate synthase (TASI_1018, TEQUI_1584); 3. aconitase (TASI_0659, TEQUI_1215); 4. isocitrate dehydrogenase (TASI_0396, TEQUI_1024); 5. α-ketoglutarate dehydrogenase (TASI_0983, TEQUI_1309); 6. Succinyl-CoA synthetase (TASI_0746, TEQUI_1545); 7. succinate dehydrogenase (TASI_1020, TEQUI_1586); 8. fumarate hydratase (TASI_0400, TEQUI_1028); 9. malate dehydrogenase (TASI_1024, TEQUI_1590); 10. malic enzyme (TASI_0984, TEQUI_1308); 11. glutamate dehydrogénase (TASI_1163, TEQUI_0187); 12. pyruvate phosphate dikinase (TASI_0871, TEQUI_1418); 13. triose-phosphate isomerase (TASI_0409, TEQUI_1037); 14. fructose-bisphosphate aldolase (TASI_1324, TEQUI_0348); 15. fructose-bisphosphatase (TASI_0582, TEQUI_1137); 16. glucose-6-phosphate isomerase (TASI_1244, TEQUI_0262); 17. transketolase (TASI_1328, TEQUI_0352). Carbon sources identified as metabolized during *T.asinigenitalis* MCE3 and *T.equigenitalis* MCE9 GEN III MicoPlate™ experimentation (Table S6) are highlighted in green. A dotted line indicates that the gene is missing.

The *T. asinigenitalis* and *T. equigenitalis* genomes are predicted to have a complete TCA cycle (Figure 6) whereas the three main pathways allowing the conversion of glucose into pyruvate are missing since (i) the 6-phosphofructokinase gene of glycolysis (Embden–Meyerhof–Parnas pathway) is absent, (ii) the pentose phosphate pathway lacks both a non-oxidative branch and transaldolase, and (iii) we did not identify any gene for the Entner-Doudoroff pathway. Consistent with this observation, Biolog GEN III MicroPlateTM assays showed that only three organic acids were metabolized as carbon sources by the two species of the Taylorella genus: malate, glutamate and α-ketoglutarate (Table S6 and Figure 6). Malate and α-ketoglutarate are intermediates of the TCA cycle and glutamate after de-amination in α-ketoglutarate by glutamate deshydrogenase (11 in Figure 6) can also be incorporated in the TCA cycle. In a previous study it was reported that *T. equigenitalis* respiration was stimulated by TCA cycle intermediates malate, citrate and succinate, but not by glucose, fructose, maltose or sucrose [6]. These observations are partially consistent with our observations since no metabolic activity was detected in our experiments in the presence of citrate and succinate. These differences could be explained by strain variability, if these metabolites require specific transport system, for example, or by differences related to the experimental procedures.

From our analysis, it appears that the acetyl-CoA needed to supply the TCA cycle is mainly produced through the conversion of malate to pyruvate by the malic enzyme (10 in Figure 6) followed by conversion of pyruvate to acetyl-CoA by pyruvate dehydrogenase (1 in Figure 6). Furthermore, the compounds required for the purine, pyrimidine and lipopolysaccharide biosynthesis pathways are produced through the gluconeogenesis pathway and the partial pentose phosphate pathway (Figure 6). This being so, we assume that the few carbon sources metabolized by *T. equigenitalis* and *T. asinigenitalis*, combined with the requirement of glycolysis and partial pentose phosphate biosynthesis pathways to synthesize purine, pyrimidine and lipopolysaccharide, could partly account for the slow growth rate of *T. equigenitalis* and *T. asinigenitalis*. Moreover, although the high atmospheric concentration of CO_2_ could have several effects on cellular physiology because it alters membrane properties and probably modifies the intracellular pH, we can assume that the *T. asinigenitalis* and *T. equigenitalis* CO_2_ requirement is partly due to its utilization as a substrate for several carboxylation reactions, such as those involved in amino acid and pyrimidine biosynthesis pathways [31].

Gene content analysis of the main general metabolism pathway of *T. asinigenitalis* and *T. equigenitalis* did not reveal any differences in gene composition, suggesting that the slower growth rate of *T. asinigenitalis* compared to *T. equigenitalis* [12] is due more to a difference in gene expression, enzyme efficiency or substrate supply than to the lack of a metabolic pathways.

### Transport systems

The uptake and efflux of solutes are mediated by transport systems embedded in the plasma membrane [32]. These membrane components feed by using their cognate high-affinity periplasmic solute-binding proteins that scavenge their specific ligand(s) [33]. To date, three solute importer families have been identified: ABC transporters (for ATP-binding cassette) [34], TRAP transporters [35], and TTT (for tripartite tricarboxylate transporters) [36]. In T. asinigenitalis, transporter systems consist of 116 genes: 80 genes encode components of ABC transporter systems (of which 72 encode 20 complete ABC transporter systems), 12 encode TRAP transporters, five encode TTTs and 19 encode miscellaneous proteins. In T. equigenitalis, transporter systems consist of 122 genes: 88 genes encode components of ABC transporter systems (of which 78 encode 21 complete ABC transporter systems), 10 encode TRAP transporters, five encode TTTs and 119 encode miscellaneous proteins (Table S7). The three carbon sources metabolized by both Taylorella species (glutamate, α-ketoglutarate and malate) could constitute substrates of several of these transporters, such as the putative glutamate aspartate ABC transport system, the putative proton/glutamate symport protein or the putative C4-dicarboxylate transport (Table S7). However, the number of transporters would suggest that *Taylorella* species are able to transport and metabolize other carbon substrates not yet identified.

Despite the reported inability of *T. equigenitalis* to utilize sugars [6], confirmed herein for both Taylorella species by Biolog GEN III MicroPlateTM assays (Table S6), we identified in *T. asinigenitalis* and *T. equigenitalis* genomes several components of the phosphotransferase system (PTS) generally involved in the transport and phosphorylation of carbohydrates. We identified genes encoding the PTS Enzyme I (TASI_1132 and TEQUI_0099), the phosphocarrier protein HPr (TASI_1133 and TEQUI_0100), one cytoplasmic protein EIIA (TASI_1134 and TEQUI_0101), one EIIA-like protein PtsN (TASI_0239 and TEQUI_0851) and one HPr kinase/phosphorylase (HprK/P) (TASI_0238 and TEQUI_0850). In the context of bacteria unable to use sugar, the presence of genes involved in PTS seems surprising. However, the lack of EIIB and EIIC homologs suggest that the PTS is not dedicated to sugar transport and phosphorylation [37] in *Taylorella* species, but rather on alternative route for other substrates. Indeed, in previous studies, PTS paralogous proteins have been shown to be involved in alternative phosphotransferase routes, including an unusual PTS-dependent utilization of dihydroxyacetone [38] and the establishment of a connection between carbon and nitrogen metabolisms [39], which could explain the presence of these genes in *Taylorella* genomes.

### Respiratory capacity

Sequence analysis of *T. asinigenitalis* and *T. equigenitalis* genomes reveals the presence of genes for the synthesis of NADH dehydrogenase, succinate dehydrogenase, cytochrome c reductase, a complete set of genes for ATP synthase, and one terminal oxidase belonging to the cbb3-type cytochrome c oxidase (Table S8). The cbb3-type cytochrome c oxidases, due to their great affinity for oxygen, are involved in microaerobic metabolisms and it has been suggested that their expression is required for the successful colonization of anoxic tissues [40]. Consequently, the cbb3-type cytochrome c oxidase may be an important determinant of the pathogenicity of *Taylorella* species.

### Oxidative stress tolerance

During *Taylorella* genome analysis, we identified for both species one superoxide dismutase (TASI_0915 and TEQUI_1375), one catalase (TASI_1292 and TEQUI_0317), one glutathione reductase (TASI_0256 and TEQUI_0384), one thiol peroxidase (TASI_0972 and TEQUI_1320), one alkylhydroperoxidase protein D (TASI_0501 and TEQUI_1062), one alkyl hydroperoxide reductase protein C (TASI_0502 and TEQUI_1063), two antioxidant thioredoxins (TASI_0667, TASI_1027 and TEQUI_1592, TEQUI_1225), two thioredoxin reductases (TASI_0294, TASI_1178 and TEQUI_0201, TEQUI_0906) and the protein-repairing peptide-methionine sulfoxide reductases MsrA/B (TASI_1464 and TEQUI_0502) [41]. This analysis shows that both species share identical set of genes involved in oxidative stress resistance, suggesting a comparable capacity to face oxidative stress. Moreover, by the number of genes identified as being involved in oxidative stress resistance, it appears that both *Taylorella* species are well equipped to face oxidative stress potentially encountered in the host.

### Secretion systems

Protein secretion is a key virulence mechanism of pathogenic and symbiotic bacteria, which makes the investigation of secreted proteins (‘effectors’) crucial for understanding molecular bacterium/host interactions [42]. Secretion systems are required to transport proteins across the cell membrane and play a role in virulence [43] and fitness [44]. During analysis of *Taylorella* genomes, we identified three secretion systems in *T. asinigenitalis* and four in *T. equigenitalis*. Table S9 shows the list of putative secretion systems identified on the basis of computational prediction in *T. asinigenitalis* and *T. equigenitalis*.

Type II secretion systems (T2SS), considered the main terminal branch of the general secretion pathway [45], are found in a wide range of Gram-negative species and are responsible for the extracellular transport of hydrolytic enzymes, toxins and other proteins crucial to the pathogenesis of many microorganisms [45], [46]. T2SS require the presence of an N-terminal signal peptide in order to utilize the Sec or twin-arginine translocation (Tat) pathways for protein translocation from cytoplasm to periplasm. During Sec-dependent or Tat-dependent secretion, proteins are first produced as precursors containing an N-terminal cleavable signal sequence. They are transported through the inner membrane via a proteinaceous complex [46]. During export, the signal peptide is cleaved by a signal peptidase and mature proteins released into the periplasmic space, then folded proteins are translocated across the outer membrane [45]. In both *Taylorella* species, we identified a complete Sec and Tat translocation pathway (Table S9), the signal peptidases SPase I required for N-terminal signal peptide cleavage of secreted proteins (TASI_0543 and TEQUI_1105) and the signal peptidase SPase II required for cleavage of lipoprotein signal peptides (TASI-1092 and TEQUI_0053). SignalP prediction of secreted proteins allowed us to identify 258 and 298 proteins (16.8% and 19.1% of total proteins respectively) potentially harboring a signal peptide and thus potentially secreted via T2SS in *T. asinigenitalis* and *T. equigenitalis* respectively (Tables S10 and S11). By their potential location at the host/pathogen interface, these proteins could constitute an interesting subject for further *Taylorella* pathogenesis studies.

In both *Taylorella* genomes, we identified a Type III secretion system (T3SS) potentially encoding a Type IVb tight adherence (Tad) pili [47] (Table S9). Considered as a ubiquitous pili-mediated host colonization and persistence mechanism [48], it has been shown that Tad pili are essential for biofilm formation, colonization and pathogenesis in numerous genera including *Haemophilus*, *Pasteurella*, Pseudomonas and Yersinia [47]. These data are consistent with the previously reported observation of pili expressed in vivo in *T. equigenitalis* [8].

Found in both Gram-positive and Gram-negative bacteria as well as in some archaea, T4SS is used for the transport of macromolecules such as proteins and DNA across the cell envelope [49]. T4SS can mediate the transport of monomeric proteins, multi-subunit protein toxins and nucleoprotein complexes [50]. Most T4SSs used to transfer proteins are found in pathogenic bacteria, where they play an important role in virulence by, for example, establishing pathogen/host interaction and/or transferring toxic effector proteins or protein complexes into the host cell's cytoplasm [50]. In *T. equigenitalis*, we identified nine T4SS-related proteins (VirB1, VirB4, VirB5, VirB6, VirB8, VirB9, VirB10, VirB11 and VirD4 (Table S9). The overall structure of the *T. equigenitalis* T4SS region seems to be related to a cytotoxin-associated gene (cag) pathogenicity island found in highly virulent Helicobacter pylori strains [50] which forms a syringe-like pilus structure to inject virulence factors such as the CagA effector protein into host target cells [51]. The lack of T4SS in *T. asinigenitalis* could partly explain the difference in virulence capacity between the two *Taylorella* species.

Structural analysis of the recently-identified type VI secretion system (T6SS) components [52] suggests that this secretion system mimics a bacteriophage machinery which punctures target cell membranes and translocates effector proteins in host cells. Representing a novel mechanism of delivering soluble effectors [53], T6SSs contribute to the virulence development of various pathogens and are often activated upon contact with target cells [53], but may also foster commensal or mutualistic relationships between bacteria and eukaryotes or mediate cooperative or competitive interactions between bacteria [54]. During *Taylorella* genome analysis, we identified six T6SS-related genes (Table S9) including Hcp (hemolysin-coregulated protein) and VgrG (valine–glycine repeat) that might serve as a conduit for T6SS-specific soluble effector proteins [55].

### Regulatory functions

Relatively few gene-encoding transcriptional regulators were identified in *T. asinigenitalis* and *T. equigenitalis*. A total of 31 and 30 potential transcriptional regulators were identified respectively, including for each, six two-component systems and two sigma factors (Table S12). No putative extracytoplasmic function (ECF) type σ factor was identified. These features likely reflect adaptation to a stable nutritional environment, where fewer biosynthetic functions and fewer adaptations are required [56].

### Virulence factors

As the CEM infection remains in the genital tract and does not invade or damage host tissues, it is likely that virulence-associated determinants of *T. equigenitalis* are involved in factors related to attachment to the host, such as extracellular matrix, host cells, or to intercellular adhesion [57] rather than damaging tissues. In keeping with this observation, we identified in silico several genes potentially involved in binding and the colonization of host cells based on homology with known microbial virulence factors and automated genome-wide screening for virulence-associated motifs [58]. We did not identify enzymes which cause damage to host tissues such as hyaluronidase or hemolysins. Of the genes common to both species and identified as potentially involved in binding and colonization of host cells, we identified the abovementioned O-antigen encoding genes, genes encoding for proteins containing ankyrin [59] and a Sel1 subtype of the tetratricopeptide repeat motif (TPR/SEL1) [60]. Proteins encoding these eukaryotic domains have been shown to be of importance in the interaction of various intracellular bacterial pathogens with their eukaryotic host cells [61], [62]. Within the *T. asinigenitalis* genome, we identified six TPR/SEL1 repeats containing proteins (TASI_0026, TASI_0290, TASI_0331, TASI_0362, TASI_0643 and TASI_0687) and one ankyrin protein (TASI_0860). In the *T. equigenitalis* genome, we identified five TPR/SEL1 repeats containing proteins (TEQUI_0900, TEQUI_0945, TEQUI_0979, TEQUI_1197 and TEQUI_1243) and two ankyrin proteins (TEQUI_1429, TEQUI_1600).

Of the *T. equigenitalis*-specific genes identified as potentially involved in binding and colonization of host cells, we can cite the abovementioned hemagluttinin-related proteins, the three putative efflux system transmembrane RND proteins, T4SS and the YadA and Hep_Hag domains containing proteins (Table 2 and Table S1). We also identified among the T. *equigenitalis*-specific CDSs randomly inserted into the genome, virulence-associated genes for TonB-dependent lactoferrin and transferrin receptors (TEQUI_0057, TEQUI_0058, TEQUI_0902 and TEQUI_0903) and for the Heat shock protein 60 (Hsp60: TEQUI_0973). TonB-dependent outer membrane receptors for lactoferrin or transferrin are identified as allowing iron to be imported from the mammalian iron carriers lactoferrin and transferrin across the outer membrane [63]. Iron being essential for microbial growth, the ability to acquire ferric iron from the host is directly related to virulence, which suggests that the more virulent character of *T. equigenitalis* could be due to a better iron acquisition capacity than *T. asinigenitalis*. Hsp60 has been characterized in particular in *Legionella pneumophila* as a multifunctional chaperonin that can be expressed on the bacterial cell surface and act as an invasion factor for non-phagocytic cells, or be released into the host cell and act as an effector capable of altering organelle trafficking, the organization of actin microfilaments and cell signaling pathways [64]. The absence of this protein in *T. asinigenitalis* suggests that cell-invading capacity is specific to *T. equigenitalis*.

During *T. asinigenitalis* genome screening we did not identify any *T. asinigenitalis*-specific genes potentially involved in virulence that were absent from *T. equigenitalis*.

## Discussion

Little is known at the molecular level about the mechanisms of *Taylorella* pathogenicity. Neither the virulence mechanism nor host resistance are known. The first comparative genomic analysis of the two members of the *Taylorella* genus presented above revealed a close relationship between the two species. The limited size of *Taylorella* genomes and their conserved synteny suggest quite a strong selection pressure and a good adaptation to their ecological niche. The fact that the main genetic differences are concentrated in a few rearrangement loci mainly linked with mobile genetic elements (e.g. bacteriophage, rearrangement hot spot, R/M) suggests restricted adaptation driven by mobile elements rather than a need to adapt to diverse environments. These observations appear consistent with the conserved metabolic pathways and the strict nutritional requirements of these two species. In this context, we can hypothesize that horse infections arise more from contamination through contact with animals displaying asymptomatic carriage of *Taylorella* species [4] than from an unidentified environmental source of contamination. Indeed, these bacteria lack the catabolic pathways required to survive in many other environments, and it seems unlikely that *Taylorella* species are able to propagate outside of the host.

From the perspective of the intracellular facultative lifestyle of *T. equigenitalis* [10] and putatively of *T. asinigenitalis*, we could assume that the slow growth rate and small number of metabolized carbon sources—despite the usual availability of a large variety of carbon sources in mammalian host cells—is due to a “filtered” nutrient supply [65] allowing survival at the expense of slow growth. Indeed, in order to survive as long as possible within the infected host cells, bacteria should not withdraw too many of the basic nutrients essential for host cell metabolism or their host will soon starve to death and the bacteria quickly lose their protective niche [65].

The chromosomal inversion across the replication axis between *Taylorella* strains generating a characteristic X-shaped symmetrical DNA dot plot (data not shown), has already been reported for numerous bacteria [66], [67], [68], and seems frequent during bacterial evolution. Although the function of such inversions is often not determined, it has been suggested that it could maintain the distortion of the replichore induced by the insertion of foreign genetic elements and/or generate genetic shuffling to create a novel gene pool that can enhance virulence and environmental fitness [68].

The fact that the majority of strain-specific genes are located within specific regions generally associated with atypical GC content (Figures 1 and 4) suggests that these regions were incorporated into the genome during a recent horizontal gene transfer, and not had time to decay to the genome-wide average GC content. Moreover, given that these bacteria share the same in vivo niche, we may consider that the genital Equidae microbiome constitutes the source of these horizontally-transferred genes.

The screening of systems that mediate resistance to infection by foreign DNA genetic material showed that the *T. asinigenitalis* genome seems to be more efficiently equipped to defend itself against phage integration or prophage integration (with one CRISPR system and one R/M system) than *T. equigenitalis*, which can defend itself by only one R/M system. Despite this, a prophage was only detected in the T. *asinigenitalis* genome and not the *T. equigenitalis* genome. This difference in phage and phage-resistance genes content have to be further confirmed in other strains of each species in order determine if these feature are representative at the species level and may therefore have a meaning in term of phage interaction and impact on gene transfer potential.

We identified numerous secretion systems in *Taylorella* genomes: complete T2SS, T3SS and T6SSs were identified for both strains and T4SS was identified only in the *T. equigenitalis* genome. Protein secretion being a key virulence mechanism of pathogenic bacteria, crucial in the pathogenesis of molecular bacterium/host interactions, it would be of great interest to study these systems. The absence of T4SS in *T. asinigenitalis* could partly explain the difference in virulence capacity between the two *Taylorella* species. The other two main characteristics that could explain the difference in virulence capacity between T. asinigenitalis and *T. equigenitalis* are (i) a potentially better capacity of *T. equigenitalis* to acquire ferric iron from the host, revealed by the presence of lactoferrin and transferrin receptors in *T. equigenitalis*, absent from *T. asinigenitalis* and (ii) the presence in *T. equigenitalis* alone of an Hsp60 homolog, a protein that can be expressed on the bacterial cell surface and act as an invasion factor [64]. Moreover, although virulence factors are generally considered to be associated with host interactions and pathogenicity, it should be noted that many of them can also be considered fitness factors in a non-virulence context [69]. Adhesins, for example, are important for colonizing all manner of niches, although colonization does not necessarily lead to infection and disease, which can be illustrated by the asymptomatic carriage of *T. equigenitalis* and *T. asinigenitalis* by many mares, as previously reported [4], [11].

Given the close overall similarity between genome sequences, a future functional investigation of species-specific CDSs would appear to be of interest, in particular with respect to inter-species differences in terms of growth ability, animal host preference and pathogenic capacity (Additional files 1 and 2). In this context, it would be beneficial to experimentally determine whether *T. asinigenitalis*, like *T. equigenitalis*, is able to invade and replicate in cultured cells [10] in order to concentrate pathogenic studies on genes belonging to one or both species.

The sequencing of these two related species that share similar lifestyles provides insights into the biology of these organisms, notably by refining and expanding our knowledge of their phylogeny, metabolism and virulence. This comparison of *T. equigenitalis* genomes led to the identification of several species-dependent genes. Studying these regions will help us to clarify intrinsic differences in the *Taylorella* genus, and potentially their host specificity. Our virulence-associated factors analysis suggests that *T. equigenitalis* is more efficiently equipped than *T. asinigenitalis* in terms of pathogenicity-related factors, but further investigation is required to determine whether it would be justified to declare CEM caused by *T. asinigenitalis*. Now that both *Taylorella* genomes have been sequenced and annotated, it appears necessary to develop molecular genetics tools including transformation of *Taylorella* in order to develop functional studies on the mechanisms involved in its virulence.

## Materials and Methods

### Bacterial strains and culture conditions

The *T. asinigenitalis* strain was isolated in 2004 from the genital tract of a 6-year-old donkey jack from a stud farm in the Loir-et-Cher, France. After identification, the strain was maintained by the French National Reference Laboratory for CEM (ANSES, Dozulé laboratory for equine diseases, France).

*T. asinigenitalis* and *T. equigenitalis* strains were inoculated on ready-to-use chocolate agar media (AES Chemunex, Combourg, France) and plates were incubated at 37°C in 7% (v/v) CO_2_ in air for 72 h and 48 h respectively. Genomic DNA was extracted as previously described [70]. Biolog GEN III MicroPlate™ assays (Biolog Inc., http://www.biolog.com) were performed according to the manufacturer's recommendations. Microplates were incubated 24 h at 37°C in 7% (v/v) CO_2_ in air before results were analyzed.

### Sequencing and assembly

Whole-genome sequencing of *T. asinigenitalis* entailed a combination of GS FLX [71] and Solexa paired-end sequencing technologies [72] (carried out by Beckman Coulter Genomics, Danvers, MA, United States). Genomic libraries containing 3-kb inserts were constructed and 342,191 reads (including 21.5% of paired–end reads) were produced using the GS FLX system, giving 57-fold coverage of the genome, then assembled into four large contigs in one potential large-scale scaffold using Mira software [73]. A total of 1.5 million reads with an average length of 92 bp were generated using an Illumina Solexa Genome Analyzer II and mapped to the contigs using the consed software [74] to correct any errors generated by the 454 technology. The order and orientation of the four large contigs were confirmed by PCR and assembled into a single sequence. Protein similarities with the KEGG protein database were used for KEGG orthology and pathway assignment [75].

### Annotation

Annotation resulted from merging the results obtained from the RAST (Rapid Annotation using Subsystem Technology) server [76], tRNAscan-SE-1.21 [77], and RNAmmer-1.2 [78] followed by manual curation. The overall subsystem category distributions of *T. asinigenitalis* and *T. equigenitalis* genomes were similar (Figure S1). CRISPR loci were detected with CRISPRFinder [79]. Secreted proteins were identified by Effective analysis (http://effectors.org) [42]. The *T. asinigenitalis* genome sequence has been deposited in the EMBL/GenBank database under accession no. CP003059. The accession numbers of the genome sequences used for comparative analyses are listed in Table S13.

### Analysis of the occurrence of *T. asinigenitalis* and *T. equigenitalis* large genomic inversion by PCR

The orientation of the large chromosomal inverted region between *T. asinigenitalis* and *T. equigenitalis* chromosomes depicted in Figure 4 was examined with the LA-PCR method (Takara, Otsu, Japan) using site-specific primer pairs (Table S3) in 30 T. *asinigenitalis* strains inclusive and 30 *T. equigenitalis* strains inclusive (Table S4). Briefly, PCR was performed as follows: 94°C for 1 min for one cycle, 98°C for 10 sec and 68°C for 7 min for 30 cycles, and 72°C for 10 min for one cycle. The amplified fragments were separated using 0.7% agarose gel electrophoresis and visualized by ethidium bromide staining.

### Electron microscopy

*Taylorella* cultures were rinsed in PBS (pH 7.2), fixed with 2.5% glutaraldehyde in cacodylate buffer 0.1 M pH 7.4 overnight at 4°C. During fixation, the cells were sedimented on Thermanox® coverslip coated with poly-L-lysine. The cells were rinsed in cacodylate buffer 0.2 M pH 7.4 in the presence of 0.2 M Sucrose and post-fixed 1 hour with 1% osmium tetroxyde in cacodylate buffer 0.1 M pH 7.4 in the presence of 0.1 M sucrose (at 4°C protected from light), The cells were rinsed in cacodylate buffer 0.2 M pH 7.4 in the presence of 0.2 M Sucrose. The cells were then dehydrated in a progressive bath of ethanol (70–100%) and critical point dried (CPD 030 LEICA Microsystem). The cells were sputtered with platinum and observed with a JEOL 6400F scanning electron microscope (JEOL Ltd, Tokyo, Japan).

## Supporting Information

Figure S1 **Subsystem distribution of CDSs in** ***T. asinigenitalis*** **MCE3 and T.** ***equigenitalis*** **MCE9.** The RAST subsystem-based annotation successfully categorized 53% of the predicted coding sequences of *T. asinigenitalis* MCE3 into 254 subsystems and 53% of *T. equigenitalis* MCE9 into 256 subsystems. Overall, subsystem category distribution of *T. asinigenitalis* MCE3 and *T. equigenitalis* MCE9 are similar, with some variations including RNA metabolism, cell walls and capsules and amino acids and derivatives.
(DOCX)

Table S1 ***T. equigenitalis*** **MCE9 specific CDSs.** Genes with no homolog in *T. asinigenitali*s MCE3 by reciprocal FASTA using a minimum cutoff of 50% amino acid similarity over 80% or more of the sequence.
(XLSX)

Table S2 ***T. asinigenitalis*** **MCE3 specific CDSs.** Genes with no homolog in *T. equigenitalis* MCE9 by reciprocal FASTA using a minimum cutoff of 50% amino acid similarity over 80% or more of the sequence.
(XLSX)

Table S3 **Primers used for orientation analysis of the large genomic inversion region in** ***Taylorella*** **strains.**
(DOCX)

Table S4 ***Taylorella*** **strains used in the study of large genomic inversion.**
(DOCX)

Table S5 **Characteristics of CRISPR loci found in** ***T. asinigenitalis*** **MCE3 and sequence similarities of CRISPR spacers.** Spacer similarities were determined by BLASTN against the NCBI Nucleotide collection. No description in “origin” or “BLAST E-value” indicates that no similarity was found in the database.
(XLSX)

Table S6 **Results of Biolog GEN III (MicroPlateTM) growth assays** ***T. asinigenitalis*** **MCE3 and** ***T. equigenitalis*** **MCE9.**
(XLSX)

Table S7 **List of predicted transport systems in** ***T. asinigenitalis*** **MCE3 and** ***T. equigenitalis*** **MCE9.**
(XLSX)

Table S8 **List of genes potentially involved in** ***T. asinigenitalis*** **MCE3 and** ***T. equigenitalis*** **MCE9 respiratory capacity.**
(XLSX)

Table S9 **Predicted gene composition of secretion systems in** ***T. asinigenitalis*** **MCE3 and** ***T. equigenitalis*** **MCE9.**
(XLSX)

Table S10 ***T. asinigenitalis*** **MCE3 secreted proteins according to SignalP prediction.**
(XLSX)

Table S11 ***T. equigenitalis*** **MCE9 secreted proteins according to SignalP prediction.**
(XLSX)

Table S12 **Putative transcriptional regulators and two-component signal transduction systems identified in both** ***Taylorella*** **species.**
(XLSX)

Table S13 **GenBank accession numbers and taxon numbers of genomes used for phylogenetic analysis.**
(DOCX)
