# Supplementary material for: Genomic Characterization of the Taylorella Genus
Source: PLoS One. 2012 Jan 3;7(1):e29953. doi: 10.1371/journal.pone.0029953 (PMC3250509; doi:10.1371/journal.pone.0029953)
Supplement: Figure S1 — Subsystem distribution of CDSs in T. asinigenitalis MCE3 and T. equigenitalis MCE9. The RAST subsystem-based annotation successfully categorized 53% of the predicted coding sequences of T. asinigenitalis MCE3 into 254 subsystems and 53% of T. equigenitalis MCE9 into 256 subsystems. Overall, subsystem category distribution of T. asinigenitalis MCE3 and T. equigenitalis MCE9 are similar, with some variations including RNA metabolism, cell walls and capsules and amino acids and derivatives. (DOCX) [file pone.0029953.s001.docx]

**Figure S1 Subsystem distribution of CDSs in *T. asinigenitalis* MCE3 and T. *equigenitalis* MCE9**. The RAST subsystem-based annotation successfully categorized 53% of the predicted coding sequences of *T. asinigenitalis* MCE3 into 254 subsystems and 53% of *T. equigenitalis* MCE9 into 256 subsystems. Overall, subsystem category distribution of *T. asinigenitalis* MCE3 and *T. equigenitalis* MCE9 are similar, with some variations including RNA metabolism, cell walls and capsules and amino acids and derivatives.
