# Supplementary material for: Genomic Characterization of the Taylorella Genus
Source: PLoS One. 2012 Jan 3;7(1):e29953. doi: 10.1371/journal.pone.0029953 (PMC3250509; doi:10.1371/journal.pone.0029953)
Supplement: Table S3 — Primers used for orientation analysis of the large genomic inversion region in Taylorella strains. (DOCX) [file pone.0029953.s004.docx]

**Table S3 Primers used for orientation analysis of the large genomic inversion region in *Taylorella* strains.**

| **Strain** | **Primer** | **Sequence (5’ to 3’)** | **Position** | **Description^a^** | **Lenght (bp)** |
| --- | --- | --- | --- | --- | --- |
| *T. asinigenitalis* MCE3 | L499 | TCCCCAGTTGAACTTTAGCC | 1025722 | Upstream of region 8 |  |
|  | L501 | AAGACTGCCATAAGTCGACG | 731468 | Downstream of region 7 | 5841 |
|  | L502 | ACGCAAAATCATAGGACGCG | 725646 | Upstream of region 7 |  |
| *T. equigenitalis* MCE9 | L504 | ATCAAGCTTCATCTCCTCCC | 1658254 | Upstream of region 8 |  |
|  | L505 | GGCGATCAAGTAATGCAAGC | 1341034 | Upstream of region 7 | 5389 |
|  | L506 | TGTGCTACCTGTCTTTCTCC | 1346404 | Downstream of region 7 |  |

^a^ Numbered regions refer to the Figure 4
