# Supplementary material for: Genomic Characterization of the Taylorella Genus
Source: PLoS One. 2012 Jan 3;7(1):e29953. doi: 10.1371/journal.pone.0029953 (PMC3250509; doi:10.1371/journal.pone.0029953)
Supplement: Table S4 — Taylorella strains used in the study of large genomic inversion. (DOCX) [file pone.0029953.s005.docx]

**Table S4** ***Taylorella* strains used in the study of large genome inversion**

| **Bacterial species** | | **Strain No.^1^** | **Isolation**  **year** | **Sampling site or origin** | **Country** | **Isolate source** | **Age (year)** |
| --- | --- | --- | --- | --- | --- | --- | --- |
| ***T. asinigenitalis*** | | |  |  |  |  |  |
|  | MCE2 | | 2004 | - | France | Jack | 6 |
|  | MCE3 | | 2004 | - | France | Jack | 6 |
|  | MCE4 | | 2004 | - | France | Donkey | - |
|  | MCE5 | | - | - | France | Jack | - |
|  | MCE6 | | 2005 | - | France | Jack | 6 |
|  | MCE10 | | 2005 | Urethral fossa | France | Jack | 4 |
|  | MCE11 | | 2005 | Urethral fossa | France | Jack | 10 |
|  | MCE12 | | 2005 | Urethral fossa | France | Jack | 15 |
|  | MCE13 | | 2005 | Urethral fossa | France | Jack | 5 |
|  | MCE14 | | 2005 | Urethral fossa | France | Jack | 3 |
|  | MCE20 | | 2004 | - | France | Jack | - |
|  | MCE21 | | 2004 | - | France | Jack | - |
|  | MCE22 | | 2004 | - | France | Jack | - |
|  | MCE23 | | 2004 | - | France | Jack | - |
|  | MCE24 | | 2004 | - | France | Jack | - |
|  | MCE46 | | 2001 | - | France | Jack | - |
|  | MCE47 | | 2001 | - | France | Jack | - |
|  | MCE73 | | 1999 | - | France | Jack | - |
|  | MCE76 | | 1999 | - | France | Jack | - |
|  | MCE86 | | 2001 | Clitoridia sinus | France | Mare | - |
|  | MCE124 | | 1998 | - | France | Jack | - |
|  | MCE235 | | 1995 | Urethral fossa, urethra and sperm | France | Stallion | - |
|  | MCE265 | | 2005 | Urethral fossa | France | Jack | 2 |
|  | MCE266 | | 2005 | Urethral fossa | France | Jack | 6 |
|  | MCE473 | | 2006 | Urethral fossa | France | Jack | 7 |
|  | MCE474 | | 2006 | Urethral fossa | France | Jack | 5 |
|  | MCE475 | | 2006 | Urethral fossa | France | Jack | 5 |
|  | MCE479 | | 2006 | Urethral fossa | France | Stallion | 10 |
|  | MCE497 | | - | ATCC 700933 | USA | - | - |
|  | MCE513 | | - | Veterinary laboratories agency | UK | - | - |
| ***T. equigenitalis*** | | |  |  |  |  |  |
|  | MCE1 | | 2005 | Sheath | France | Stallion | 4 |
|  | MCE8 | | 2005 | Urethral fossa | France | Stallion | 4 |
|  | MCE9 | | 2005 | Urethral fossa | France | Stallion | 4 |
|  | MCE15 | | 2005 | Urethral fossa | France | Stallion | 6 |
|  | MCE16 | | 2005 | Clitoridia sinus | France | Mare | 8 |
|  | MCE17 | | 2005 | Clitoridia sinus | France | Mare | 8 |
|  | MCE25 | | 2005 | - | France | - | - |
|  | MCE26 | | 2005 | - | France | - | - |
|  | MCE27 | | 2005 | - | France | - | - |
|  | MCE28 | | 2005 | - | France | - | - |
|  | MCE29 | | 2004 | - | France | - | - |
|  | MCE30 | | 2004 | - | France | - | - |
|  | MCE31 | | 2004 | - | France | - | - |
|  | MCE32 | | 2004 | - | France | - | - |
|  | MCE33 | | 2004 | - | France | - | - |
|  | MCE34 | | 2004 | - | France | - | - |
|  | MCE35 | | 2004 | - | France | - | - |
|  | MCE36 | | 2004 | - | France | - | - |
|  | MCE37 | | 2004 | - | France | - | - |
|  | MCE38 | | 2005 | Urethral fossa and urethra | France | Stallion | 9 |
|  | MCE39 | | 2005 | Clitoridia sinus | France | Mare | 6 |
|  | MCE40 | | 2002 | - | France | - | - |
|  | MCE41 | | 2002 | - | France | - | - |
|  | MCE42 | | 2002 | - | France | - | - |
|  | MCE43 | | 2001 | - | France | - | - |
|  | MCE44 | | 2001 | - | France | - | - |
|  | MCE49 | | 2001 | - | France | - | - |
|  | MCE50 | | 2001 | - | France | Donkey | - |
|  | MCE52 | | 2005 | - | France | - | - |
|  | MCE54 | | 2001 | - | France | - | - |
|  |  | |  |  |  |  |  |

NOTE. MCE is the name of the *Taylorella* strains collection of our Contagious Equine Metritis Group.
