# Supplementary material for: Genomic Characterization of the Taylorella Genus
Source: PLoS One. 2012 Jan 3;7(1):e29953. doi: 10.1371/journal.pone.0029953 (PMC3250509; doi:10.1371/journal.pone.0029953)
Supplement: Table S13 — GenBank accession numbers and taxon numbers of genomes used for phylogenetic analysis. (DOCX) [file pone.0029953.s014.docx]

**Table S13** GenBank accession numbers and taxon numbers of genomes used for phylogenomic analysis.

| Species | Accession no. | TaxID |
| --- | --- | --- |
| *Achromobacter xylosoxidans* A8 | CP002287 | 762376 |
| *Acidovorax citrulli* AAC00-1 | CP000512 | 397945 |
| *Acidovorax ebreus* TPSY | CP001392 | 535289 |
| *Alicycliphilus denitrificans* BC | CP002449 | 596153 |
| *Bordetella avium* 197N | AM167904 | 360910 |
| *Bordetella bronchiseptica* RB50 | BX470250 | 257310 |
| *Bordetella parapertussis* 12822 | BX470249 | 257311 |
| *Bordetella pertussis* Tohama I | BX470248 | 257313 |
| *Bordetella petrii* DSM 12804 | AM902716 | 340100 |
| *Burkholderia cenocepacia* MC0-3 | CP000958, CP000959, CP000960 | 406425 |
| *Burkholderia mallei* SAVP1 | CP000525, CP000526 | 320388 |
| *Burkholderia multivorans* ATCC 17616 | AP009385, AP009386, AP009387 | 395019 |
| *Burkholderia thailandensis* E264 | CP000085, CP000086 | 271848 |
| *Burkholderia xenovorans* LB400 | CP000270, CP000271, CP000272 | 266265 |
| *Comamonas testosteroni* CNB-2 | CP001220 | 688245 |
| *Cupriavidus metallidurans* CH34 | CP000352 | 266264 |
| *Delftia acidovorans* SPH 1 | CP000884 | 398578 |
| *Herbaspirillum seropedicae* SmR1 | CP002039 | 757424 |
| *Herminiimonas arsenicoxydans* | CU207211 | 204773 |
| *Janthinobacterium sp.* Marseille | CP000269 | 375286 |
| *Leptothrix cholodnii* SP-6 | CP001013 | 395495 |
| *Methylibium petroleiphilum* PM1 | CP000555 | 420662 |
| *Polaromonas naphthalenivorans* CJ2 | CP000529 | 365044 |
| *Polynucleobacter necessarius* subsp. *necessarius* STIR1 | CP001010 | 452638 |
| *Ralstonia solanacearum* PSI07 | FP885891, FP885906 | 859657 |
| *Rhodococcus equi* 103S | FN563149 | 685727 |
| *Rhodoferax ferrireducens* T118 | CP000267 | 338969 |
| *Taylorella equigenitalis* MCE9 | CP002456 | 937774 |
| *Taylorella asinigenitalis* MCE3 | CP003059 | 1008459 |
| *Thiomonas intermedia* K12 | CP002021 | 75379 |
| *Variovorax paradoxus* EPS | CP002417 | 595537 |
| *Verminephrobacter eiseniae* EF01-2 | CP000542 | 391735 |
